# Influence of the Distribution of Tag IDs on RFID Memoryless Anti-Collision Protocols

**DOI:** 10.3390/s17081891

**Published:** 2017-08-17

**Authors:** Nikola Cmiljanic, Hugo Landaluce, Asier Perallos, Laura Arjona

**Affiliations:** 1 DeustoTech—Fundación Deusto, Avda. Universidades, 24, 48007 Bilbao, Spain; hlandaluce@deusto.es (H.L.); perallos@deusto.es (A.P.); laura.arjona@deusto.es (L.A.); 2 Faculty of Engineering, University of Deusto, Avda. Universidades, 24, 48007 Bilbao, Spain

**Keywords:** RFID, anti-collision, tag identification, memoryless protocols, collison tree, window, ID distribution, RFID tag ID

## Abstract

In recent years, Radio Frequency Identification (RFID) has become very popular. The main feature of this technology is that RFID tags do not require close handling and no line of sight is required between the reader and the tags. RFID is a technology that uses radio frequencies in order to identify tags, which do not need to be positioned accurately relative to the reader. Tags share the communication channel, increasing the likelihood of causing a problem, viz., a message collision. Tree based protocols can resolve these collisions, but require a uniform tag ID distribution. This means they are very dependent of the distribution of the IDs of the tags. Tag IDs are written in the tag and contain a predefined bit string of data. A study of the influence of the tag ID distribution on the protocols’ behaviour is proposed here. A new protocol, called the Flexible Query window Tree (FQwT) is presented to estimate the tag ID distribution, taking into consideration the type of distribution. The aim is to create a flexible anti-collision protocol in order to identify a set of tags that constitute an ID distribution. As a result, the reader classifies tags into groups determined by using a distribution estimator. Simulations show that the FQwT protocol contributes to significant reductions in identification time and energy consumption regardless of the type of ID distribution.

## 1. Introduction

Radio Frequency Identification (RFID) technology is one of the best and most popular technologies. It uses radio waves to automatically identify people or objects. In 2015, over 10.1 billion RFID tags were produced, and this figure will rise to 19 billion by 2026 [1]. One of its most important features is its ability to identify objects wirelessly without being in its line-of-sight, as opposed to bar code technology [2]. This proves to be beneficial in many industries, such as, health care, retail, inventory management, supply-chain management, and wireless sensor networks [3,4]. The main aim of RFID is to reduce logistical overhead, cost, and minimize product losses. It provides a process with higher productivity.

RFID originated from the radar technology, and it has greatly evolved since then. Currently, this technology goes beyond identification purposes, and it is being used in localization [5,6] and sensing [7] applications. Furthermore, there is a growing class of battery-free computational and sensing tags which go beyond simple barcodes replacement functionality, named Computational RFID (CRFID) and RFID sensor tags [8].

RFID systems consist of the following components: the reader, the tags, and a back-end database. The reader is a control unit with one or more antennas. This device interrogates the tags to transmit their data. Every RFID system contains one or more tags. Every tag has a unique identification code (ID) and includes an integrated chip and an antenna and can be attached to objects. Some tags may contain an integrated battery and they are called active tags. Another kind are passive tags, that do not contain a battery: their power is supplied by the reader. The back-end database is intended to store and further process the data about the identified tags, in a database [9].

The reader sends out electromagnetic waves which the tag antenna receives and backscatters with its ID, converting the waves to digital data. In every slot of an interrogation round, the reader attempts to read a single tag from among a population of many. When more than one tag simultaneously transmits to a reader, this leads to the cancellation of communication and the resulting message is illegible. This tag state is called a ‘collision’ [10,11]. Collision results in a loss of identification time and an increase in power consumption, deteriorating the performance of RFID applications. To mitigate the influence of the tag collision problem, RFID readers use an anti-collision protocol. Protocols that are implemented in order to reduce the influence of collisions can be sorted into three groups: Aloha based, Tree based, and Hybrid protocols. Aloha based protocols are probabilistic since the tag responses are organized randomly [12,13,14]. Tree based protocols are known as deterministic [15,16]. All the tags in the interrogation area will be read within a certain time limit. Hybrid protocols have been created in order to mitigate the problems of the Aloha and Tree based protocols. They increase the manufacturing costs of their complex reader and tag designs [17,18].

All the information about the tags is stored in an RFID electronic chip. The information contained in a tag’s electronic chip depends on its application. This designation is called the tag ID and presents a unique identifier (UII, Unique Item Identifier, or EPC code) [19]. The standardization of the tag IDs with the EPC standard provides an improvement of RFID, allowing it to access global networks. To satisfy these needs, RFID is increasingly demanding larger tag IDs [19]. Once this ID has been written in the electronic chip, it can be read and, in some tag solutions, can be changed. The main requirement for the standard EPC is that all tags set in the interrogation area must have a unique ID. The tag IDs are not always uniformly distributed. Consequently, different tag ID distributions are considered in the present paper. The main problem in some anti-collision protocols is that their behaviour directly depends on the type of the distribution and on the ID length [20]. These protocols, in the RFID environment where the bits in the tag ID are randomly organized, suffer a loss in the consumed bits, latency, consumed energy, and many other parameters. This happens in Tree-Based protocols because the tags’ answers directly depend on a reader request. In a heterogeneous environment, many protocols do not know how to skip some unnecessary queries and avoid a huge number of collisions. This problem does not concern Aloha protocols, because the tags respond with 16-bit random numbers, which are independent of the tag ID. In the literature, most protocols are tested under a uniform or homogeneous distribution [21]. A uniform distribution (UD) is organized in such a manner that the tag ID is 100% variable. This means that almost all protocols work with a certain number of tags that use the left and right sides of the binary tree uniformly. However, in real RFID systems, the tag IDs are randomly organized in the interrogation area.

This paper presents a study of how the tag ID distribution influence tree based memoryless protocols with the focus at the Media Access Control (MAC) layer level. Memoryless protocols do not require any counter or memory to identify a group of tags. In this kind of protocol, the tags do not need to store information to be identified. In order to solve the problem presented in this paper, there will be proposed the Flexible Query window Tree (FQwT) protocol. FQwT has been created with the aim of improving the flexibility in adaptation to different tag ID distributions. The rest of the paper is organized as follows. Section 2 presents the previously related literature, including the Query Tree (QT), Query window Tree (QwT), and Smart Trend Traversal (STT) protocols. Representation of the ID Distribution are given in Section 3. In Section 4, the proposed FQwT is presented. Section 5 presents simulation results and the analysis of the influence of the tag ID distribution on the memoryless protocols presented in state of the art. Finally, Section 6 concludes this paper and presents avenues for future research.

## 2. Background

This section presents an overview of the existing anti-collision protocols. Subsequently, some related work will be analysed.

First, some basic terms are given, in order to properly understand the most closely related tree based protocols:

- Slot: The period of time that divides the tags’ responses is called a slot. It includes a reader command and a tag response. During the identification process, and depending on the number of tag responses received by the reader, three types of slot can occur: collision, idle, and success. A collision occurs when more than one tag answers the reader’s command in the same slot. When no tag responds to the reader’s command, then an idle slot happens. Finally, a success occurs when just one tag is correctly read by the reader and, therefore, identified.
- Query: A bit-string broadcast command transmitted by a reader. The query consists of a prefix-binary string that all tags in the interrogation area will compare with their ID. In case a tag’s ID does not match the query, the reader command will be rejected.
- Identification process: This is the time period that includes a certain number of time slots or rounds that the reader needs to identify all tags in the range of its antenna.

### 2.1. Multi-Access Methods

Each anti-collision protocol uses some multi-access method for identification in order to physically separate the transmitters’ signals. Accordingly, they can be categorized into four different categories: Space Division Multiple Access (SDMA), Frequency Division Multiple Access (FDMA), Code Division Multiple Access (CDMA), and Time Division Multiple Access (TDMA) [2].

SDMA: Protocols based on this method can point the beam to different areas in order to identify tags. The channel is spatially separated using complex directional antennas. Another way of achieving this is by using multiple readers. This method is pretty expensive and requires a complex antenna design [2,22].

FDMA: Tags transmitting in one of several different frequency channels requiring a complex receiver at the reader. However, this technique is expensive and is only intended for some particular applications [2].

CDMA: Requires tags to multiply their ID by a pseudo-random sequence (PN) before transmission. Unfortunately, this method consumes a lot of power and can be classified as a group with elevated demands [2,22].

TDMA: As it is less expensive, this method is the most widely used. The transmission channel is divided between the participants, which ensures that the reader can identify tags at different times in order to avoid interfering with another. The characteristics of the distribution in space of the tags are not considered. This method results in the same tag’s being read more than once. The majority of the existing protocols are based on TDMA [2].

In an RFID environment, anti-collision protocols typically use the TDMA method [11]. These protocols can be divided into three categories: Aloha-based protocols, which are probabilistic; tree-based protocols, which are deterministic; and hybrid protocols, which use a combination of the previously referred to methods.

### 2.2. Aloha-Based Protocols

Aloha-based protocols use a random-access strategy in order to successfully identify the number of tags in an interrogation area. They belong to the category of probabilistic protocols because the tags transmit their own IDs in randomly selected slots in a frame in order to reduce the possibility of a collision. However, it is not guaranteed that all the tags will be identified in the process of interrogation. Every frame consists of a certain number of slots. In case of a collision, the tags will be invited to transmit their data again, with a random time delay. Aloha-based protocols can be divided into three subgroups: Slotted Aloha (SA), Frame Slotted Aloha (FSA), and Dynamic Frame Slotted Aloha (DFSA). In SA, the time is divided into several slots and each tag must randomly select a slot in which they will transmit their data. The communication between the reader and the tag is based synchronously. In FSA, all tags need to transmit the data within a frame of fixed length. In order to improve on this, DFSA has been developed. This protocol can mitigate the disadvantage incurred by FSA, through changing the frame size according to an estimate of the number of tags. At the beginning of each frame, the reader informs the tags about the frame length *F*. Every tag selects a random number *n* in the range [0,F−1], and responds in the *n*th slot. At the end of the frame, the reader estimates the number of colliding tags, then adjusts *F* accordingly. This protocol has the well-known tag starvation problem, in the sense that a tag might not be correctly read during a reading cycle. Tag estimation can involve some disadvantages, such as: increasing the computational costs in the identification process; errors that degrade the efficiency of the protocol; and in some protocols, only the initial frame can be set by the tag number estimation.

### 2.3. Tree-Based Protocols

One of main features of this kind of protocol is that they are deterministic and all tags in the interrogation area are identified during the process. In this anti-collision protocol, a reader will interrogate all tags for the next bit of their ID. If two different binary values are received from the population of tags, the reader will be able to detect the collision. These protocols have tags with a simple design, and work very well with a uniform set of tag IDs, but are slower than Aloha-based protocols [23].

Tree based protocols have a muting capability, and all the tags in the communication process can be silenced after identification [11]. The following is a list of some of the tree-based protocols: Query Tree (QT), QwT (Query Window Tree), and Smart Trend Transversal (STT). All of these protocols are deterministic and read all the tags in the interrogation area under the assumption of a non-impaired channel communication.

### 2.4. Hybrid Protocols

Hybrid protocols combine the advantages of tree-based and Aloha-based protocols to avoid their problems and to provide a better tag identification. Most of them first implement a tree-based procedure and a tag estimation procedure in order to predict the number of tags. Subsequently, a combination of the procedures of the Aloha-based protocol and a tree-based protocol reduces the identification time. This kind of protocol can significantly improve the performance in comparison with previous ones. A recent suggestion was Tree Slotted Aloha (TSA) [24] and Binary Tree Slotted Aloha (BTSA) [25]. TSA uses a tree structure, and the tags’ responses are organized in slots, as in FSA. All new frames are applied on collided tags. These procedures require complex tags, and have a problem with starvation the same as Aloha-based protocols. In the BTSA protocol, the tags randomly choose a slot after a reader query. If a collision occurs, a tree-based procedure is employed to solve the problem and to identify the tags. This protocol requires an initial estimation of the frame.

### 2.5. Query Tree Protocol

The query tree protocol (QT) is one of the most representative memoryless protocols, where the reader must provide the tags with a query *q* and the matching tags must respond with their full ID [23]. The tag response directly depends on the current query, ignoring the past history of communication. QT tags have only simple hardware requirements, because they only compare the reader query with their own ID and respond if it coincides. The identification process consists of more rounds, in which the reader sends a query, and tags whose ID prefixes match the current query respond with their whole ID binary value. In case of a collision, the reader forms two new queries by appending *q* with a binary 0 or 1. New queries will be placed in a Last Input First Output stack (LIFO). If there is no answer upon a query, the reader knows that there is no tag with the required prefix, and the query is rejected. In case just one tag responds to the reader query, that tag will be identified. By extending the query prefixes until only one tag’s ID matches, the algorithm can identify the rest of the tags. The identification procedure is completed when the LIFO stack is empty.

**Figure 1:**
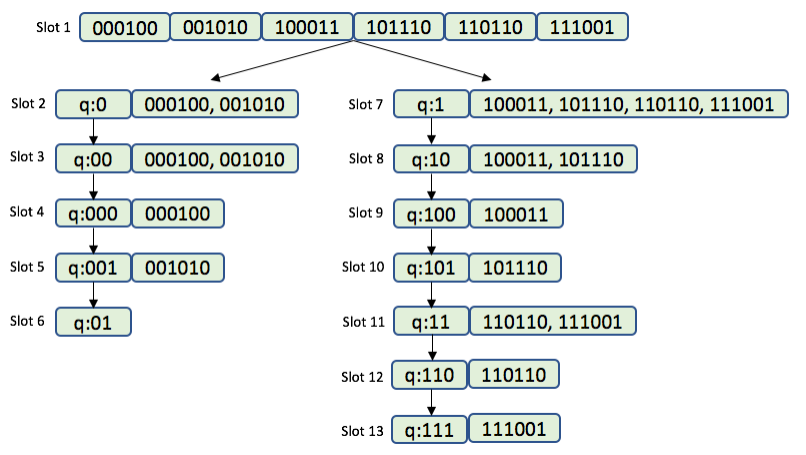
Example of the QT protocol.

Figure 1 shows the QT protocol being used to read 6 tags. Each tag uses an ID of length k=6 bits. At the beginning, the LIFO stack is empty and the reader starts with a null string. After a collision occurs, the reader pushes queries 0 and 1 into the LIFO stack. During the second round, the reader pops from the stack and transmits query 0. In the example in the Figure 1, tags 000100 and 001010 match the required prefix, which causes both to transmit and collide. The reader is unable to understand the messages from the tags. The reader then pushes into the stack the queries 01 and 00. In the next round, the reader transmits query 00. Again, both tags respond with their ID, and a new collision occurs. In the stack, the following new queries are added, 001 and 000. The reader transmits query 000 and only one tag responds (000100). This tag is successfully identified and will not answer any later reader requests. The reader then transmits query 001 in slot 4, which matches tag 001010. In the next round, the reader pops and transmits query 01. For this query, there will be no response since there are no tags with that prefix. In slot 7, the reader transmits query 1 and the tag from the right side of the tree responds. Four tags will receive this query and a new collision occurs. The reader experiences a collision, since tags 100011, 101110, 110110 and 111001 responded to the query 1. As a result, queries 11 and 10 are pushed onto the stack. The identification process is repeated until round 13, in which the reader transmits the last query (111) from the stack. Overall, the reader used 13 rounds to read 6 tags.

### 2.6. Smart Trend Traversal Protocol

The Smart Trend Traversal protocol (STT) is a deterministic and memoryless protocol, created with the aim of reducing the number of collisions in the QT protocol [21]. This protocol has the ability to dynamically issue queries according to an online learned tag density and distribution. It proposes a combination of the QT protocol and the shortcutting method in order to skip a query which results in collision. When the protocol detects the potential possibility of a collision, it will avoid it and move to the bottom level of the binary query tree. STT provides trend recognition. The reader keeps track of the tag density and distribution in order to issue the subsequent queries, and consequently, maintains at a minimum the number of empty slots and collision slots. In this protocol, it is not necessary to have any prior knowledge of the network, and it outperforms the existing protocols. The ideal number of queries can be the total number of single nodes. The ideal queries group, referred to as the query traversal path (QTP), is denoted by $Q=q_{1},q_{2},q_{3},\cdots ,q_{n}$, where $q_{n}$ is the last query used in the identification process. It is difficult to achieve, but it is desirable to get close to its value. The reader can calculate the subsequent queries depending on the tag response, which can be classified into three types:

- A collision occurs when QTP is at too high level and should move down by adding a longer prefix to the query. Consequently, the reader appends *t* bits of 0’s to the last query, where *t = s + $n_{col}$*− 1. Let *s* denote the minimum increase, and $n_{col}$ be the number of consecutive colliding slots.
- An idle slot occurs when no tag responds to a reader query. QTP needs to traverse up just one level, which can lead to a new collision. This rule will be applied only to the right side. If the empty response comes from the left side of the tree, QTP must move horizontally to the right. The reader will decrease the query length by *m* bits, where *m = s + $n_{emp}$*− 1 and $n_{emp}$ is the number of consecutive idle slots.
- Upon a successful response, a single node is visited, indicating that the tag has been identified successfully by the reader. Then QTP moves to the symmetric node if the query finishes with 0, but it returns one level if the query finishes with 1.

**Figure 2:**
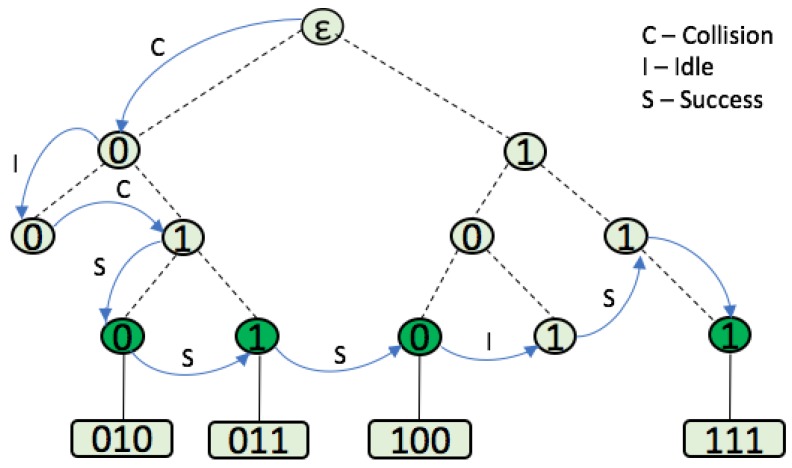
Example of the STT protocol.

The identification process of the STT protocol, which was explained above, is depicted in Figure 2 with 4 tags. In conclusion, STT significantly reduces the number of collisions, the identification time, and the energy consumption, compared to the existing Aloha-based and tree-based protocols.

### 2.7. Window Method and Query Window Tree Protocol

**Figure 3:**
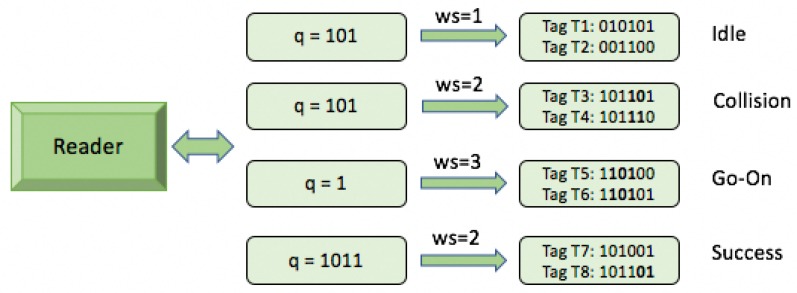
Window synchronized answer.

In the majority of tree-based protocols, tags respond with their full ID or with the bits of the last query, when the query sent by the reader matches the tag ID prefix. In order to reduce the number of bits transmitted by the tag, the window method is proposed [26]. In the identification process, a lot of slots end up colliding, and this contributes to a huge waste of bits. Protocols that use the window method reduce the number of bits transmitted by the tags. The window is defined as the bit-string of length *ws* bits transmitted by a tag in a slot. This bit-string is computed at the reader side, respecting the condition *0 < ws < k*. It is shown in Figure 3. Most tree based protocols use a fixed tag response during the identification process, but some protocols use different methods based on an operational process with a dynamic response that is based on window synchronization. The Query window Tree (QwT) is the first protocol that uses this method [26].

The QwT protocol is a memoryless tree based protocol that applies a dynamic bit window to QT. Tags respond directly, depending on the current query. QwT tags compare their ID value with the received query and transmit a certain bit string managed by the reader. This reduces the passive tags’ complexity, energy, and identification time.

When tags appear in the interrogation area, the reader will transmit a query of length *L* bits. Tags will respond if their ID prefix matches the query sent by the reader, but with a previously specified number of bits. One of the main features of QwT is that the total number of collisions is decreased by transforming potential collisions into partially successful slots. This is a new type of slot, called a go-on slot. The previously explained window method is implemented in the QwT protocol. The window allows tags to transmit just a bit-string instead of their full ID. If a tag’s ID matches a reader query, it will synchronously transmit the adjacent bits ws of the ID. This protocol uses cyclic redundancy check (CRC) bits in order to differentiate between the types of tag responses. Accordingly, the slot types that can occur in the QwT protocol can be classified into four groups:

- Collision slots: When the reader cannot differentiate the answer the reader will create two new queries by appending ‘0’ and ‘1’ on the former query [*q${}_{1}$, q${}_{2}$, …, q${}_{L}$*]. The window size *ws* will remain unchanged from the previous query.
- Idle slots: When there is no response, the reader will discard the query and will retain the same *ws* from the last command.
- Go-on slots: When at least one tag responds with *ws* bits and the reader is able to understand it. If the equation ID *L + ws < k* is not true, the reader will transmit a new query created from the former query and received window. On this query, the reader will append an updated *ws*.
- Success slots: This is a type of go-on slot where the reader successfully receives the last part of the tag ID and *L + ws = k*. Then the reader saves the tag ID, calculates the new *ws*, and continues with the identification process.

Using the QwT protocol, the reader computes *ws* using (1), where β is an adjustable parameter. This heuristic function is presented in order to provide dynamism to the value of *ws*. Additionally, the heuristic function is used to recalculate the window size *ws = f(L)*. It is applied to go-on slots, since during a collision, *ws* will be held unchanged. The proposed protocol provides a decrease in the number of tags transmitting bits, but increases the number of slots and readers transmitting bits. Altogether, this protocol achieves significant energy savings and a reduction in identification time.

(1) $$\begin{array}{cc} f\left(L\right)=k\left(1-e^{-\beta L}\right),\; & 0<L\leq k \end{array}$$

## 3. Representation of the ID Distribution

**Figure 4:**
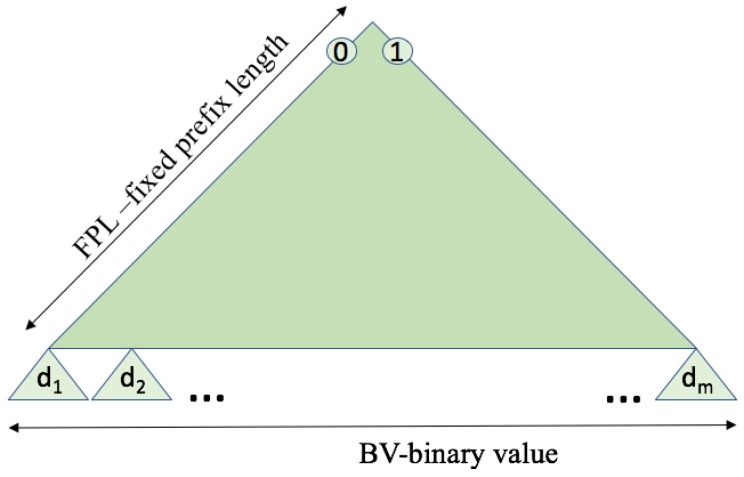
Types of tag ID distribution.

The whole set of tag IDs sets up a distribution of binary strings with the shape of a binary tree, starting from the most significant bit (MSB) to the least significant bit (LSB). Almost all protocols from the literature have been created and tested under UD [15,16,23,26]. The behaviour of some protocols particularly depends on the distribution that they use. UD is the special type of ID distribution that has constant probability for the organization of the bits in the set of tag IDs. The tag IDs are structured in a binary tree, in which each node has at most two children, which are referred to as the left child and the right child. For the UD, the tag ID bits in the tree data structure are distributed with equal probability on the left and right sides of the tree. In real RFID systems, it is unrealistic to assume that the tag ID distribution is always uniform. Consider the goods in a supermarket, which belong to several specific categories. Different tag ID distributions can greatly influence the performance of a protocol. Figure 4 clearly shows a three-cornered binary tree with potential variations, where the organization of the distribution depends on changing the following parameters:

- Fixed prefix length (FPL) defines a specific organization of the distribution where all tags in the interrogation area share the initial part of the ID, of that length.
- Binary value (BV) denotes the horizontal position of the tag ID distribution at an FPL level. This horizontal position is given as a percentage of 2${}^{FPL}$− 1.
- The number of the uniform subdistributions ($d_{m}$) asumes the organization of the tag ID distribution in several subdistributions *m* following UDs. The higher the number of subdistributions, the more similar the main distribution will be to a UD.

**Figure 5:**
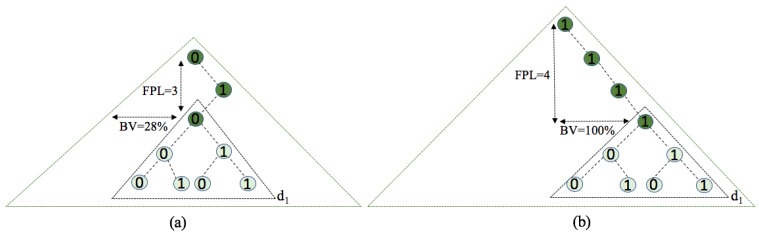
Examples of tag ID distribution types for $d_{1}$ and adjustable values: (**a**) FPL = 3; BV = 28%; (**b**) FPL = 4; BV = 100%.

An example of verying FPL and BV, under fixed $d_{m}$, is shown in Figure 5. If FPL = 3, BV = 28%, and $d_{1}$, the first tag in this binary structure will have the initial binary value 010. BV is calculated as the percentage value from the largest value in the binary tree for the used FPL (111). In another case, when FPL = 4 and BV = 100%, the tags will fix four bits for their initial parts and the tag ID sets will start with the fixed four bit value 1111. Therefore, different ID distributions are considered in this paper. Every type of distribution will have the same number of tags but be organized with many variations.

The influence of different tag ID distribution can degrade deterministic protocols behavior, resulting in a higher energy consumption, an increased number of collisions, and a prolonged identification time. In order to solve the aforementioned problem, this paper proposes a protocol that estimates the ID distribution and further controls the protocol’s behaviour, providing flexibility to work under different ID distributions, yet with similar performances in terms of latency, energy consumed, and total bits transmitted.

## 4. Flexible Query Window Tree Protocol

This paper proposes a novel Flexible Query window Tree (FQwT) and it analyses its flexibility in detail. This protocol employs the window method and an ID distribution estimator. It is also a memoryless protocol, since the tags do not need to save information in order to be identified, and the tags’ responses directly depend on the current query and *ws*.

Excessive collisions increase energy consumption, wasting a large number of tag transmitted bits. In this paper, the FQwT protocol is presented to manage the length of the tags’ responses, in order to reduce the energy wastage in scenarios where tags have different ID distributions. FQwT has the ability to estimate the ID distribution and reduce the number of the bits in the tags’ responses, which provides significantly better behaviour regardless of the type of ID distribution followed by the tags. Consequently, this approach results in decreased: energy consumption, number of transmitted bits, and identification time.

A decrease in the number of transmitted bits in an heterogeneous tag environment is achieved by estimating the tag ID distribution and subsequently adjusting *ws* in the reader command. The window method provides the protocol to aggressively advance through the common parts of the IDs.

**Figure 6:**
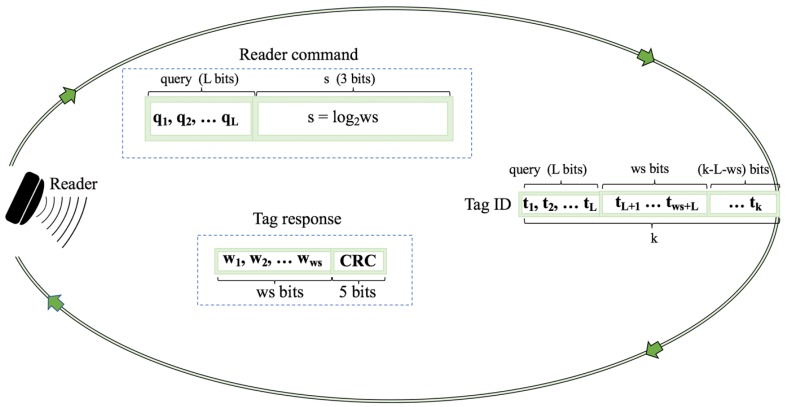
Format of the reader command and tag responses of the FQwT protocol.

The reader calculates *ws* as an integer in the range of 1 < *ws* < k but then it is transmitted, together with a query, to the tags in changed form *s* (2). The string *s* is a standardized fixed 3-bit value that the reader must send in every interrogation round. As it is shown in Figure 6, the proposed FQwT protocol sends a query and *s* to all the tags in the interrogation zone. The tags calculate *ws* using (3) from the received reader command, differentiating it from the query by using the last 3 bits of the reader command. The tags receive and compare the query with their ID; matching tags respond exclusively with the bits specified by *ws*. Plus, when the reader receives the tags’ responses, it checks what kind of slot it has received, then estimates the tag ID distribution and calculates the next query and *ws*. The reader primarily estimates the tag ID distribution. Depending on the environment, the reader can locate the organization type of the ID distribution. The protocol functioning is divided into two phases: the estimation of the distribution and the identification process.

(2) $$\begin{array}{c} s=log_{2}\mathrm{ws} \end{array}$$

(3) $$\begin{array}{c} \mathrm{ws}=2^{s} \end{array}$$

### 4.1. Phase 1: Estimation of the ID Distribution of the Tags

In order to obtain good results, the reader will, in the initial phase of the tags identification, estimate the type of distribution, store the obtained data, and use it in the subsequent operations. The first phase ends when the first tag is identified.

**Figure 7:**
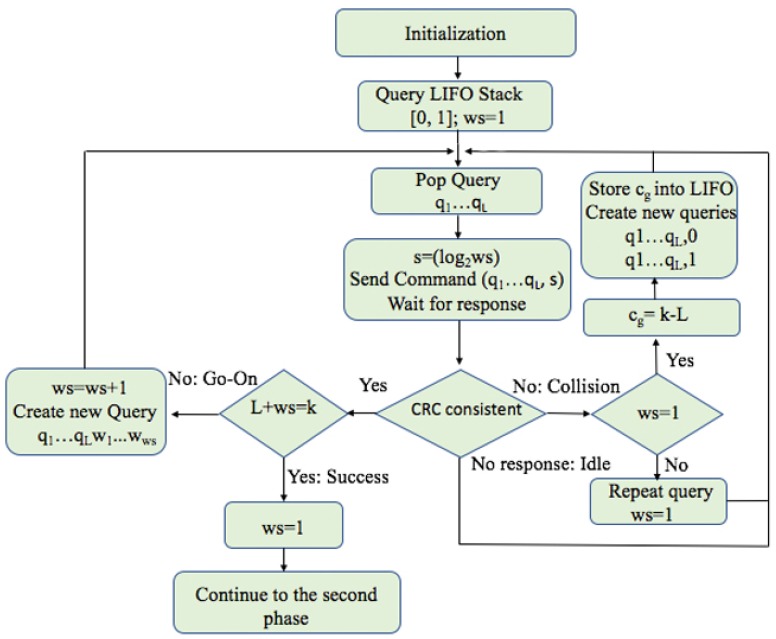
Flow chart of the first phase in the proposed FQwT protocol.

The reader flow chart of the initial procedures, called the ID distribution estimation, in FQwT is given in Figure 7. The reader initializes the procedure by pushing two new queries into a LIFO stack and then the reader starts the identification by popping the first query from the stack and transmitting it to the tags in the interrogation area. Initially, *ws* = 1. In the next interrogation rounds in the first phase, *ws* will be calculated using the same rules predefined just for this phase. Apart from the query of length *L* in the reader command, the length of *ws* is attached in the form of (2). Matching tags will calculate the value of *ws* (3) from the received string *s*. It will be the final value of *ws*.

The procedure for calculating *ws* will be used during both phases: the estimation of the distribution and the identification of the tag sets. In contrast, the calculation of *ws* will be different, depending on the phase. Also, all responding tags attach CRC bits so that the reader can differentiate the type of tag response by checking the consistency of the CRC. When the reader receives one or more responses from the tags, it will check its consistency. Depending on the type of response, the reader will act as follows:

- Upon a collision, the reader will check the value of *ws*. If *ws* is bigger than 1, it will restart at the beginning value (*ws* = 1). When *ws* = 1, the reader will calculate $c_{g}$, the difference between the ID length *k* and the current query length *L*, and locate the first group in a new type of ID subdistribution. The reader stores the first value of $c_{g}$ into LIFO and continues with the interrogation. All $c_{g}$ values are stored into the LIFO stack together with the the corresponding query, and used when a specific group is identified. Later, when the reader pops a query from the LIFO, it will use the same $c_{g}$ value for the whole identified group to calculate *ws*.
- In case of an empty response, the reader will continue the process with a new query from the stack and ws will be held unchanged.
- A go-on slot is received if the reader understands the response but the ID is not complete (*q* + *ws* < *k*). The reader will increase *ws* by 1 and store the received window in the stack and use it in the next query.
- Finally, when the reader receives last window bits and completes the whole ID, a successful slot occurs. The reader saves this tag and completes the first phase. The following procedure will follow a new phase with addition calculation.

**Figure 8:**
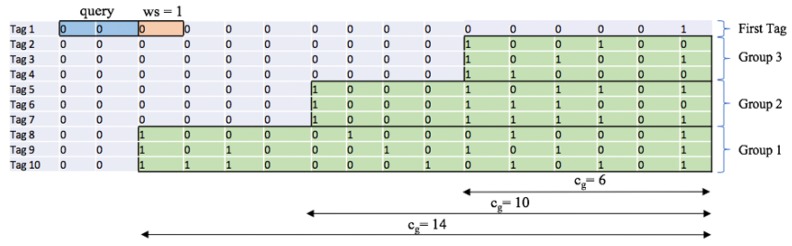
An example of the procedure of the estimation of the tag ID distribution.

**Figure 9:**
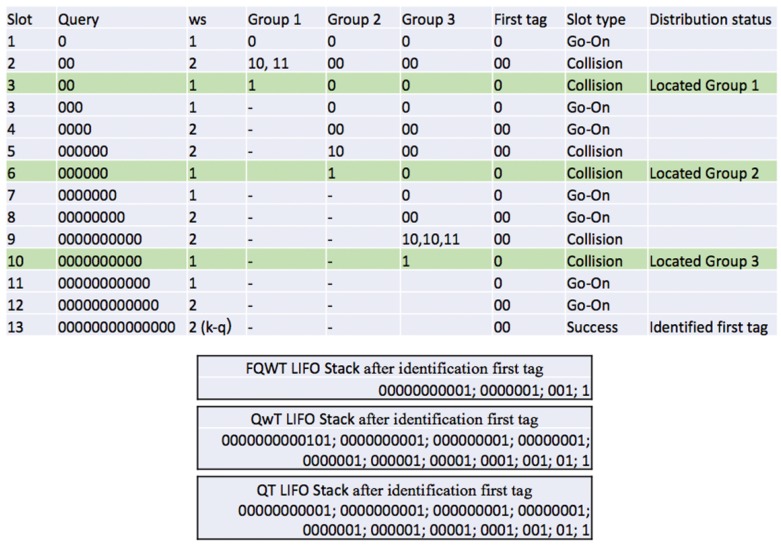
Comparison of FQwT, QwT, and QT protocols during the identification of the first tag.

An example of ID distribution estimation in an environment of 10 tags using FQwT is shown in Figure 8 and Figure 9. The initial *ws* is 1 and the ID length *k* is assumed to be 16 bits. The reader starts with a query 0. All tags from the example respond and a go-on slot occurs. A new query is created by appending the window to the last query. The reader recalculates *ws* (*ws = 2*) and *s*, and attaches it to the query. On the new reader query (00), 7 tags will answer (Tag1–Tag7) and a collision occurs. In the subsequent interrogation round, the reader will repeat the same query but with decreased *ws*. In this slot, when a collision is detected and *ws* = 1, the reader can locate the first group (Group 1) in an unknown type of tag ID distribution and store $c_{g}$ value in LIFO. From this example, the first branch is denoted by Group 1 and there are three tags (Tag8–Tag10). Subsequently, the reader checks the value of *ws*, reduces it, and creates two new queries by appending 0 and 1 (000 and 001). The same procedure is followed and Groups 2 and 3 are located and information about them will be stored into the stack. After the last query (00000000000000), only one tag responds and transmits the last part of the ID. With this step, the estimation procedure is completed. The reader located 3 groups in the interrogation area and identified the first tag ID. With this, the first phase of the process is finished. For comparison, Figure 9 presents unused queries from the stack, from FQwT, QwT and QT. The novel protocol will continue with 22 bits from the stack, QwT needs 68 bits, while the QT reader needs to spend 66 bits in order to identify the rest of the tags. Finally, when the first tag is identified, the first phase is completed and then the reader can continue with the second phase.

After the last query, the first phase is completed and the reader is able to calculate the values of FPL and $d_{m}$. In the mentioned example, the presented RFID environment has three identified groups of tags with different values of $c_{g}$, and the reader will update the number of subdistributions to $d_{3}$. Also, in this example, the reader will allocate values for FPL to 2, 6, and 10. Using this data, the majority of the binary tree can be constructed. The conclusion is that the array of $c_{g}$ after the first phase is in direct relation to FPL and $d_{m}$.

### 4.2. Phase 2: Identification Phase

**Figure 10:**
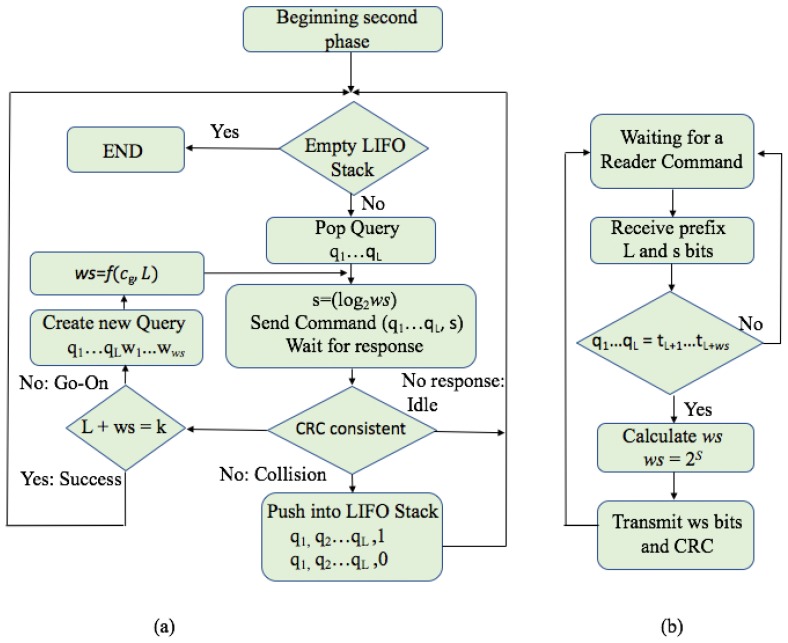
Flow chart of the proposed FQwT protocol: (**a**) for reader; (**b**) for tags.

Figure 10 shows the flow chart of the second phase. This phase is based on the identification of all tags in the interrogation zone by using the estimated values ($c_{g}$) from the first phase. An exponential heuristic function that links $c_{g}$ and *L* to *ws* (here as $f(c_{g},L)$) is presented (4) in order to provide dynamism to the value of *ws*. This function is a heuristic proposed to provide better results for the adjustment of the window size when the distribution of the ID is not homogeneous and will make a balance by reducing the tag transmitting bits and limiting the number of go-on slots. If the value of $c_{g}$ is higher than *L*, the reader will adjust *ws* to *$c_{g}$-L*. The influence of changing *ws* can only be applied to the success slots, since during a collision, or a go-on slot, *ws* will be held unchanged. This function is adjusted with a value of the parameter β = 0.5, preselected to decrease the energy consumed by the proposed protocol. In the example, explained above, the first group has $c_{1}$ = 2. This value changes according to the state in the identification process.

(4) $$\begin{array}{c} f\left(c_{g},L\right)=\frac{c_{g}}{L}k\left(1-e^{-\beta L}\right),\;0<L\leq k \end{array}$$

The process of identification for the second phase of the proposed FQwT protocol is depicted in Figure 10 in the form of a flow chart and described subsequently. The second phase starts by broadcasting a reader query, [*$q_{1}$…$q_{L}$*] of length *L*. In each round, the reader calculates the number of bits (*ws*) with which the tags must respond to a matching query, and converts it to the 3-bit string *s* which will be attached to the reader command.

**Figure 11:**
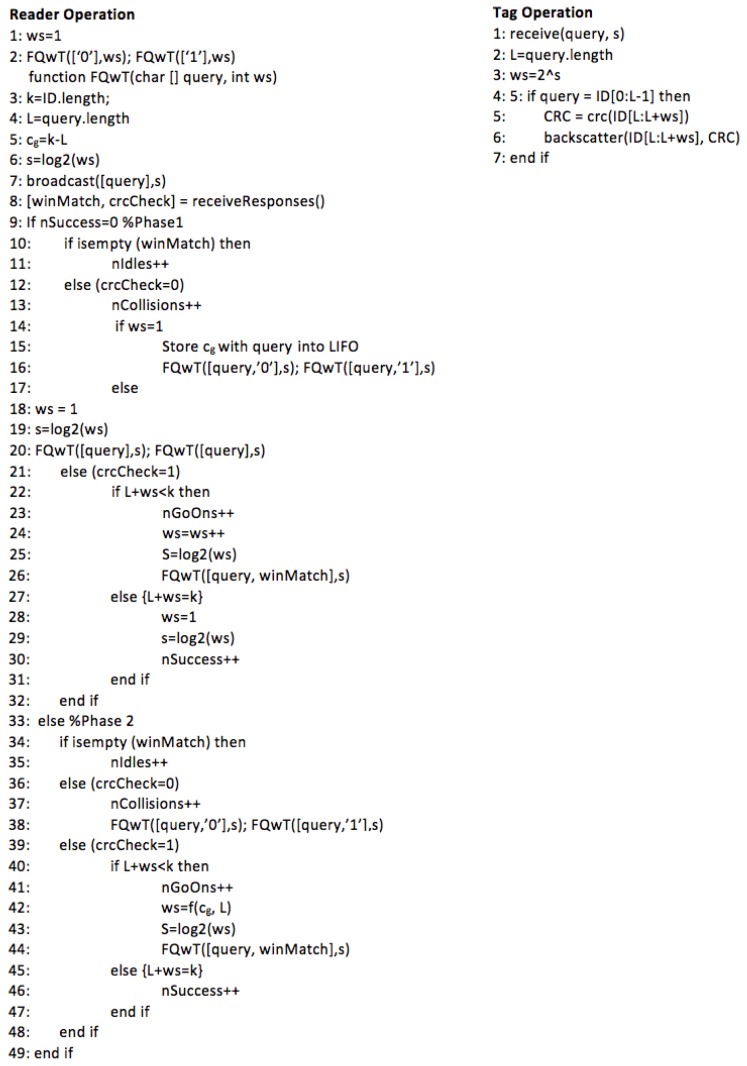
Pseudo-code of FQwT. First the reader operation is presented, then the tag operation.

Pseudo-code for the FQwT reader and tags is shown in Figure 11 for better clarity. Once the protocol begins, the reader transmits a query with appended value *s*, upon which 4 possible slot statuses can happen after a tag response: idle, collision, go-on, and success slot. In an idle slot, the reader will reject the last transmitted query and will pop the last pushed query from the stack for a new command. The reader will keep *ws* unchanged. A collision slot is detected when at least one colliding bit is found. The reader will not change the size of the *ws* from the last query, and creates two supplementary queries [*$q_{1}$, $q_{2}$, …, $q_{L}$,* 0] and [*$q_{1}$, $q_{2}$, …, $q_{L}$,* 1]. In a go-on slot the reader creates a new query by appending the received window to the former query. The new *ws* is calculated with the heuristic function. Finally, a success slot is met when the CRC validates the received window and the reader checks that *L + ws = k*. Furthermore, the reader will pop a new query from the stack and will calculate *ws* using the proposed heuristic function, according to the current distribution branch from the stack.

## 5. Simulations

This section presents an analysis of the influence of the tag ID distribution on several state of the art protocols and on the proposed FQwT. Also, the performance of FQwT will be evaluated here, and will be compared with the behaviour of some existing tree-based protocols: STT [21], QT [23], and QwT [26]. The simulations were executed using MatLab R2016b. Their performance is compared with the aim of verifying the flexibility of FQwT in different types of ID distribution. First, the influence of changing BV in FQwT is presented in the graph. Then all the protocols from the state of the art will be compared with FQwT under changing FPL and $d_{m}$. Finally, the behaviour of all protocols under changing the type of ID distribution will be presented.

The protocols’ behaviour under all variations will be presented and carefully analysed. However, different tag ID distributions can greatly influence the protocol performance. The proposed simulation defines a scenario with one reader and a fixed number of tags, *n*. For this experimentation *n* is fixed at *n* = 1000 tags, because the effects observed in the simulations results with this set of tags are similar to that of smaller sets of tags. The length of the tag IDs *k* is fixed, 128 bits, and the CRC length is assumed to be 5 bits. The simulated responses shown have been averaged over 100 iterations for accuracy. All used parameters have been chosen to comply with the EPC standard to model the simulated scenario. Tari represents the reference time interval for a data-0 transmission and is set to the standard of 6.25 μs and influences the other parameters, T1, T2, T3, RTCal, and TRCal, in accordance with the EPC standard [19]. In Table 1 are presented the protocol data when the reader sends commands and receives the responses from the tags. In Table 2, the field *Reader Command* is different depending on the protocol simulated, and is specified for all protocols, where *L* corresponds to the length of the query. The type of distribution depends on modifying three values: FPL, the BV part, and $d_{m}$. In this section, variations of these three values will be considered and the presented protocols will be simulated under them.

### 5.1. Distribution Organization by Modification of the BV Value

**Figure 12:**
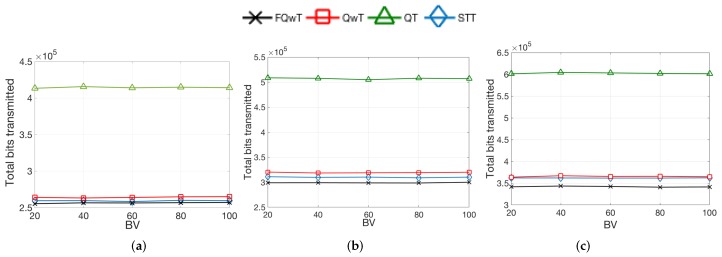
Simulation results obtained for total transmitted bits when d=1; FPL is 20, 60, and 100; and, BV varies from 20 to 100%. (**a**) FPL = 20; (**b**) FPL = 60; (**c**) FPL = 100.

This distribution organization consists of the fixed initial length determined by FPL, while the numerical value of this fixed length is presented with BV. However, BV must be calculated by using the rules of binary and decimal number systems and will vary from 0 to 100%. The simulated results in Figure 12 present the total transmitted bits under $d_{1}$. This figure shows the influence of the modification of BV on all presented protocols in terms of the total bits transmitted between the tags and the reader. The graphs show that by changing BV, the behaviour of the protocol will not be affected. In contrast, with increasing FPL, the total number of bits will increase in the identification process. These comparisons were performed with the following values of FPL: 20, 60, and 100. The simulation results for the total transmitted bits by the protocols show evidence that FQwT has a very slight improvement in total bit consumption, in comparison with the other presented protocols. The influence of varying BV produces losses in the total number of consumed bits in both protocols, STT and QwT. In contrast, QT lags behind the mentioned protocols and results in significantly increased bits consumption by reader and tags. The conclusion is that the state of the art protocols provide constant bit consumption during the modification of BV (20–100%) and the variation of this value will not have an effect on the protocols’ behaviour.

### 5.2. Distribution Organization by Modification of FPL

**Figure 13:**
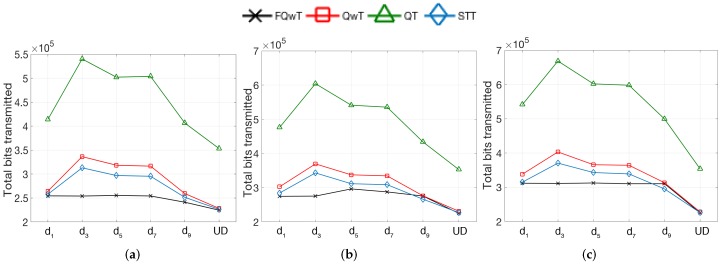
Simulation results obtained for total transmitted bits for different tag subdistributions when BV varies from 0% to 100%; FPL is 20, 60 and 100; and, $d_{m}$ varies from $d_{1}$ to UD. (**a**) FPL = 20; (**b**) FPL = 60; (**c**) FPL = 100.

Here, the different types of tag ID distribution are generated by varying FPL and $d_{m}$. The value of BV is kept fixed since from the conclusions obtained on the previous analysis, the total number of bits transmitted is not affected by this parameter. BV is assigned randomly, according to the number of subdistributions. In the case of one subdistribution, BV is randomly selected from [0, $2^{FPL}$− 1]. If the tag ID distribution is organized with more subdistributions, every subdistribution has a different BV, randomly calculated from the same range. The results will be evaluated from one subdistribution to UD. The total number of bits transmitted by each protocol from the state of the art is depicted in Figure 13, with varying FPL. Each graph shows one, fixed FPL, under more subdistributions. The tag sets are divided into a number of subdistributions and every group of subdistributions has the same initial ID part generated at the beginning of an iteration, and fixed for all the sets of tags in the identification process. The rest of the part of the tag IDs will be randomly generated. For example, in the set with 1000 tags, there are two subdistributions and FPL = 20. The first 500 tags will have the same initial part (20 bits) of the ID, while the next 500 tags will have another value in the initial part.

The performed simulations have been again parametrized using the data from Table 1, and *n* is fixed to 1000 tags. The simulated results present the total transmitted bits used in the identification process. The presented results evidence a decrease in the total bits for the proposed FQwT protocol in comparison with other protocols. By changing the number of subdistributions, FQwT preserves its flexibility. QwT and STT also prove to have good results in the UD. By increasing the number of subdistributions, their performance on the total bits transmitted will drastically increase. The variation of distribution has the greatest impact on the QT protocol on an enhanced number of subdistributions in terms of total bits transmitted. The results are not good, especially in environments with 3, 5 or 7 subdistributions.

These results indicate that FQwT significantly outperformed the presented protocols when the number of subdistribution varies from 1 to 9. This indicates that FQwT is a flexible protocol faced with changes of many distribution types, and its behaviour is not affected by a varying number of subdistributions. FQwT provides similar results as if the UD were valid. The performance of FQwT is the best when the number of subdistributions varies from 1 to 7, especially with FPL = 20. Small changes occur when FPL increases to 60, but despite these changes, it still provides good results.

**Figure 14:**
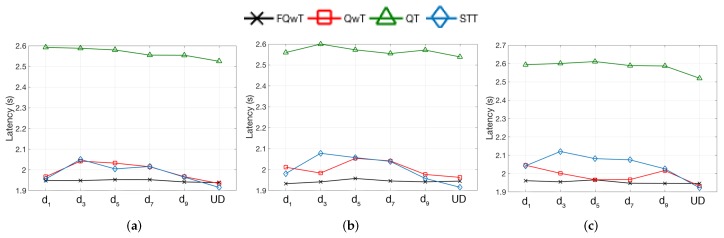
Simulation results obtained for the latency for different tag subdistributions, when FPL is 20, 60 and 100, and $d_{m}$ varies from $d_{1}$ to UD. (**a**) FPL = 20; (**b**) FPL = 60; (**c**) FPL = 100.

The time required by an RFID system to identify all tags in the interrogation zone is known as the latency. A comparison of the FQwT protocol with QT, STT, and QwT in terms of latency is presented in Figure 14. Many anti-collision protocols have been studied using the number of slots, but not the time [27]. The latency *Lat* for this simulation is calculated by using Equation (5), depending on the slot type, where $Lat_{c}$, $Lat_{s}$ (7) and $Lat_{i}$ (8), related to the tag, represent the latency during collisions, successes, and idle slots, and $Lat_{R}$ is the latency during the reader transmission.

(5) $$\begin{array}{c} Lat=Lat_{R}+Lat_{c}+Lat_{s}+Lat_{i} \end{array}$$

(6) $$\begin{array}{c} Lat_{R}=\frac{S_{RB}}{R_{DR}}+T1 \end{array}$$

(7) $$\begin{array}{c} Lat_{c};Lat_{s}=\frac{S_{TB}}{T_{DR}}+T2 \end{array}$$

(8) $$\begin{array}{c} Lat_{i}=T1+T3 \end{array}$$

Here, $S_{RB}$ represents the number of reader bits in a slot, and $S_{TB}$ the number of tag bits in each slot. $R_{DR}$ and $T_{DR}$ denote the reader and the tag data rate. On each reader command, the matching tags must respond within time T1. The reader has time T2 to receives all the transmissions, and lastly, when an idle slot occurs, the reader will wait for the tags for time T3.

**Figure 15:**
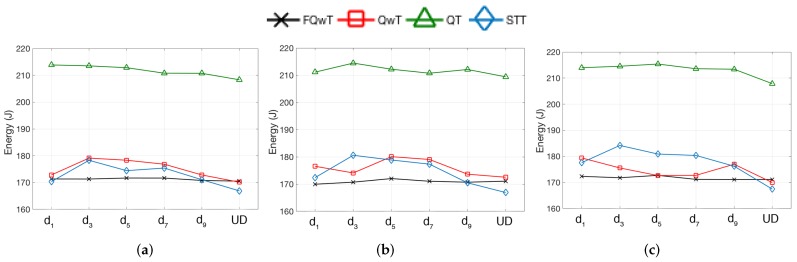
Simulation results obtained for the energy consumption for different tag subdistributions, when FPL is 20, 60 and 100, and $d_{m}$ varies from $d_{1}$ to UD. (**a**) FPL = 20; (**b**) FPL = 60; (**c**) FPL = 100.

The energy consumed by the reader is represented by *E* (9) and calculated during the time of transmitting and receiving information. In every interrogation round the reader will transmit the command to power up passive tags with power Ptx. When the reader receives a response from the tags, it will require extra power Prx. During the identification process, the total energy will be calculated by using (9), where $E_{c}$, $E_{s}$ (11) and $E_{i}$ (12) represents the energy consumed during a collision, a success, and an idle slot. $E_{R}$ (10) is the energy consumed by the reader during transmission. It is a function of the time it spends transmitting and receiving information. The comparison of the energy consumption must be made acording to the proposed expression. Figure 15 shows the energy consumption of all presented protocols. This figure shows evidence that FQwT outperforms the others in environments with 3, 5, 7, and 9 subdistributions, in terms of energy consumption. STT present a low energy consumption in all subdistributions, and the best performance is achieved in the UD. The most important observation is that FQwT is the only protocol from the comparison that provides flexibility during changes in the number of subdistributions.

(9) $$\begin{array}{c} E=E_{R}+E_{c}+E_{s}+E_{i} \end{array}$$

(10) $$\begin{array}{c} E_{R}=Ptx\left(\frac{S_{RB}}{R_{DR}}+T1\right) \end{array}$$

(11) $$\begin{array}{c} E_{c};E_{s}=\left(Ptx+Prx\right)\left(\frac{S_{TB}}{T_{DR}}+T2\right) \end{array}$$

(12) $$\begin{array}{c} E_{i}=Ptx+\left(T1+T3\right) \end{array}$$

## 6. Conclusions

This paper presented a study of how tag ID distributions can influence tree-based memoryless protocols. Several state of the art protocols have been simulated under different organizations of the ID distributions, and their results have been analysed.

A novel FQwT protocol with the ability to estimate the tag ID distribution has been presented and carefully analysed. This protocol uses the estimation results in order to calculate the ideal number of bits with which the tags must respond to queries during the interrogation round. FQwT shows the flexibility of efficient anti-collision features for RFID tag identification. The results obtained show that during a change in the number of subdistributions, FQwT keeps the flexibility features similar to that of UD. In addition, simulation comparisons showed that the FQwT is a protocol that outperforms the state of the art protocols in terms of reducing the number of transmitted bits, the latency, and increasing energy savings, and is thus to be considered as a good anti-collision solution in passive RFID systems. The proposed solution helps to improve the performance the growing number of current RFID applications, such as sensing and asset management.

## Figures and Tables

**Table 1:** Calculation of transmitted bits used in simulation.

Parameter	Value
*k*	128 bits
CRC	5 bits
Tari	6.25 μs
Data rate	160 kbps
RTCal	18.75 μs
TRCal	24.38 μs
T1	26.75 μs
T2	27.5 μs
T3	72 μs
Ptx	825 mW
Prx	125 mW

**Table 2:** Parameters used in simulations.

Protocol	Reader Command	Tag Response	Tag Response in ID Distribution Estimation
FQwT	L+3	ws+CRC	*ws*
QT	*L*	*k*	/
STT	*L*	k−L	/
QwT	$L+\lfloor \log_{2}ws\rfloor +1$	ws+CRC	/
